# Cultural beliefs that may discourage breastfeeding among Lebanese women: a qualitative analysis

**DOI:** 10.1186/1746-4358-4-12

**Published:** 2009-11-02

**Authors:** Hibah Osman, Lama El Zein, Livia Wick

**Affiliations:** 1 Department of Health Behavior and Education, Faculty of Health Sciences, American University of Beirut, Beirut, Lebanon; 2 Center for Research on Population and Health, Faculty of Health Sciences, American University of Beirut, Beirut, Lebanon; 3 Department of Social and Behavioral Sciences, Faculty of Arts and Sciences, American University of Beirut, Beirut, Lebanon

## Abstract

**Background:**

Although the health benefits of breastfeeding are well established, early introduction of formula remains a common practice. Cultural beliefs and practices can have an important impact on breastfeeding. This paper describes some common beliefs that may discourage breastfeeding in Lebanon.

**Methods:**

Participants were healthy first-time mothers recruited from hospitals throughout Lebanon to participate in a study on usage patterns of a telephone hotline for postpartum support. The hotline was available to mothers for the first four months postpartum and patterns of usage, as well as questions asked were recorded. Thematic analysis of the content of questions which referred to cultural beliefs and practices related to breastfeeding was conducted.

**Results:**

Twenty four percent of the 353 women enrolled in the study called the hotline, and 50% of the calls included questions about breastfeeding. Mothers expressed concern about having adequate amounts of breast milk or the quality of their breast milk. Concerns that the mother could potentially harm her infant though breastfeeding were rooted in a number of cultural beliefs. Having an inherited inability to produce milk, having "bad milk", and transmission of abdominal cramps to infants through breast milk were among the beliefs that were expressed. Although the researchers live and work in Lebanon, they were not aware of many of the beliefs that are reported in this study.

**Conclusion:**

There are a number of cultural beliefs that could potentially discourage breastfeeding among Lebanese women. Understanding and addressing local beliefs and customs can help clinicians to provide more culturally appropriate counselling about breastfeeding.

## Background

The health benefits of breastfeeding to both mother and infant have been well established [1,2]. In developing countries, where risks of infectious diseases and malnutrition are high, early introduction of infant formula increases the risk of serious illness that could lead to death [3]. The United Nations Children's Fund (UNICEF) has estimated that exclusive breastfeeding in the first six months of life can reduce under-five mortality rates in developing countries by 13% [4]. In addition to its health benefits, breastfeeding has significant economic and environmental benefits. Potential savings from breastfeeding in the US alone have been estimated to be around 3.6 billion dollars [2].

Exclusive breastfeeding in the first four months of life varies from 1 to 90% depending on where the baby is born [4]. This variability in breastfeeding practices is significantly influenced by cultural beliefs, socioeconomic status, ethnicity, education, urbanization, modernization, and local feeding practices [5-7]. Gender roles, social support and attitudes of friends and relatives towards breastfeeding have also been shown to affect a mother's intended duration of breastfeeding [3]. Reasons cited for early introduction of formula feeding include embarrassment, lack of social support, commercial pressures, insufficient maternity leave, and concerns about infant weight gain or breast milk quality [3].

Health care providers have an important impact on intention to breastfeed, initiation and consequent duration of breastfeeding [8]. Studies have shown that women who receive encouragement to breastfeed from health care providers are more likely to initiate and maintain breastfeeding than women who did not receive encouragement [9,10].

Cultural beliefs and local traditions are important in determining health behavior in general. Studies of feeding practices in different countries have shown a large variety of beliefs and traditions related to breastfeeding [5-7,11-16]. While some of these can encourage breastfeeding, others may discourage it. A good understanding of local beliefs, customs and traditions related to breastfeeding can help healthcare providers and breastfeeding advocates provide better support and more appropriate counselling to breastfeeding mothers.

Lebanon is a small middle-income country with a population of 4.5 million in the eastern Mediterranean. Approximately 85% of the population lives in urban centres and the fertility rate is 1.7 [17]. According to the Lebanon family health survey PAPFAM, up to 89% percent of infants are ever breastfed and the mean duration of breastfeeding is nine months [17]. A study conducted in the capital city of Beirut in 2001 showed that rates of breastfeeding were 56.3% at one month and 24.7% at four months [18]. A study of breastfeeding prevalence in Lebanon in 2005 showed that while the initiation of breastfeeding is high, exclusive breastfeeding at one month was only 52% and declines further at four and six months [19]. In that study, women cited insufficient milk, maternal or infant illness, the baby refusing the breast, and the baby being "old enough to stop" as the main reasons for stopping breastfeeding [19].

Little is known about the traditions and beliefs related to breastfeeding in Lebanon. This paper describes some cultural beliefs related to breastfeeding expressed by first-time mothers postpartum, as seen through calls to a postpartum support hotline.

## Methods

This study is part of a larger study that aimed to determine the utilization patterns of a hotline for postpartum support. The "Hotline Utilization Study" was conducted in preparation for a trial on reducing stress during the transition to motherhood (main results paper in preparation). The hotline was a mobile telephone that was answered by a midwife who was trained to respond to the questions and concerns of mothers regarding self-care, infant care, and parenting issues. Answers were driven by algorithms developed by the researchers for the study. For each call the midwife recorded information on a semi-structured data collection tool which included the caller's identification number, the time and duration of the call, the questions asked and the advice she gave the caller. All questions that the midwife deemed to require the attention of a physician were referred to the caller's physician.

Women were recruited from 17 hospitals distributed over the five regions in Lebanon between November and December of 2007. Hospitals were selected based on their volume of deliveries. The three to four hospitals with the highest number of deliveries per month were selected in each region. Study participation was limited to healthy first-time mothers who delivered at term during the study period and had no maternal or infant complications.

Eligible participants were approached by recruiters in the postpartum ward after delivery and before discharge from the hospital. After verbal consent was obtained, information about the woman was collected including intent to breastfeed, gender and birth weight of the newborn, age of the mother, and type of delivery. No identifying information was recorded. Women who agreed to participate in the study were given the number for a hotline for postpartum support. They had access to the hotline 24 hours a day for the first four months after their delivery. A study identification number was given to participants to link delivery information to calls made to the hotline. The study protocol was approved by the Institutional Research Board at the American University of Beirut. The requirement for written consent was waived since no identifying information was collected on study participants.

Soon after the initiation of the Hotline Utilization Study, a review of the telephone calls to the hotline revealed that several of the callers held beliefs about breastfeeding that were discouraging them from continuing to breastfeed their infants. The study team therefore decided to investigate this issue further by collecting information on all calls related to breastfeeding that had any reference to traditional customs or cultural beliefs for further analysis. The midwife recorded all such questions on a separate data collection sheet in colloquial Arabic using the same terms used by the caller in reference to the problem discussed.

At the end of the study, the researchers reviewed these data and analyzed the content of questions using the framework approach for qualitative research analysis [20]. Two of the three researchers reviewed the data separately and each categorized and coded questions related to beliefs and customs about breastfeeding according to theme. All three researchers then discussed these themes to resolve any differences in understanding or interpretation of the information and to validate the interpretations. Finally, our analysis was shared with the midwife to verify that our interpretation of the questions was appropriate. This paper provides an analysis of the cultural beliefs and traditions related to breastfeeding that emerged from the telephone calls.

## Results

Of the 376 women who were invited to participate in the study 353 (94%) consented. The demographic characteristics of the women enrolled are included in Table 1. At the time of enrolment, 261/353 (73.9%) of the women expressed intent to breastfeed their infants exclusively, 12 (3.4%) intended to use infant formula exclusively and 78 (22.4%) intended to combine breast and formula feeding.

**Table 1:** Characteristics of women enrolled in the study (n = 353)

**Demographic variables**	**N (%)**
Region where woman delivered	
Beirut/Mount Lebanon	125 (35.4)
South	55 (15.6)
Bekaa	70 (19.8)
North	103 (29.2)

Education	
None	6 (1.7)
Elementary	26 (7.4)
Intermediate	137 (38.8)
Secondary	76 (21.5)
University degree	108 (30.6)

Age (years)	
Less than 21	70 (19.8)
21-24	107 (30.3)
25-28	94 (26.6)
29-32	41 (11.6)
More than 32	41 (11.6)

Twenty four percent (84/353) of the women enrolled in the study called the hotline. A total of 312 calls were received during the course of the study. Fifty percent (157/312) of the calls included at least one question related to breastfeeding. Problems with breastfeeding and questions about breastfeeding technique were the most common reasons for women to call the hotline. A number of the callers who were concerned about breastfeeding described various beliefs as reasons for wanting or needing to initiate formula feeding. What follows is an elaboration on the themes that emerged during the analysis of questions related to breastfeeding.

### Quantity of breast milk

Several women wanted to introduce formula feeding because they felt their infants were not getting enough milk. These concerns were usually raised because the baby continued to cry after feedings or because the mother was no longer having the engorgement that is commonly associated with breastfeeding in the first few weeks. Women often reported that they were ready to introduce formula supplementation to satisfy the hunger of the infant.

Some of the women who were concerned about their milk supply believed that their inability to breastfeed was inherited from their maternal line. Women who held those beliefs had been told by their mothers, sisters or both that they would not be able to breastfeed successfully because this was a problem that ran in the family. Women either believed they could not produce adequate quantities of milk to sustain the needs of a newborn, or that their milk was not nutritious. We noted that women whose families held those beliefs were under significant pressure not to even attempt breastfeeding. When these women attempted to breastfeed, any fussiness from the infant or difficulties with sleep or feeding were attributed to the mother's attempt to breastfeed.

There were different concerns related to the impact on breast milk of expressing milk using a pump. A few women were afraid to express milk because they believed that expressing milk emptied the breasts and therefore decreased the quantity of breast milk. One caller believed that expressed milk was bad and should not be given to the infant.

A few women called with concerns about the effect of the evil eye on their milk supply. One of those callers referred to the "kabseh" - a belief that women who are breastfeeding can be cursed by menstruating women.

### Quality of breast milk

Several of the women in our study were concerned about harming their infants through their breast milk. Some worried that breastfeeding when they had cracked or bleeding nipples was harmful to the baby. Others were concerned about continuing to breastfeed when they had an upper respiratory tract infection or were taking medications, for fear of exposing the baby to potentially dangerous substances.

A number of women worried about the impact of their diet on the quality of their milk. They called asking about what they should and should not be eating while they were breastfeeding. Cabbage, cauliflower, and mloukhiyeh (a green leafy vegetable consumed as a stew) were commonly thought to cause bloating, gas pain, or diarrhea in the breastfeeding infant. Mothers of infants with jaundice were afraid to continue breastfeeding and in some cases were discouraged from continuing to breastfeed by their physicians.

Several mothers called the hotline for assistance to figure out whether their milk was "good" or "bad". Bad milk could be milk that was not nutritionally adequate for an infant's growth, or in the extreme cases, it could potentially "poison" the baby. Women asked about indications that would allow them to determine if their milk was good or bad, often stating that someone (usually a family member), had told them that their milk must be bad because the baby was fussy, not lasting long enough between feeds, not sleeping well or not growing adequately.

A common belief was that maternal abdominal pain could be transmitted to the infant through the breast milk and result in colic. Mothers were especially concerned about transferring their abdominal cramps to their infants if the infants were fussy.

Although most concerns were related to the mother harming her infant by having insufficient or poor quality milk, in some instances, there was some concern that the infant could harm the mother when breastfeeding. For instance, some women expressed the belief that if the baby burped while breastfeeding the mother would develop a breast infection.

## Discussion

There appear to be many beliefs and traditions surrounding breastfeeding. Analysis of the content of telephone calls to a hotline for support of first-time mothers in Lebanon revealed a number of beliefs and misconceptions that could discourage or hinder successful breastfeeding. Like many other studies, we found the quantity of breast milk that a mother produces to be a common concern and a major source of anxiety. Batal et al noted this to be the primary reason for early introduction of formula in their study on Lebanese women [19]. Researchers cited similar concerns about insufficiency of breast milk as a common reason for early discontinuation of breastfeeding in many different countries including Iran [13], Turkey [6,12], Brazil [3,21], and the United States [1], among others.

The perception of insufficient breast milk has been attributed to the mother's interpretation of the baby's crying as a sign of hunger [22]. In our population, this concern was related to both the crying of the infant, as well as the resolution of breast engorgement, which was interpreted by the mother as a sign for concern.

Interestingly, we found that family members (particularly the woman's mother) are important sources of discouragement of breastfeeding. This was noted in another study on breastfeeding in first-time mothers when women identified their own mothers, their partners and their partner's mothers as the main sources of discouragement of breastfeeding [23]. In our study population, several women were discouraged from attempting to breastfeed because they were believed to be biologically incapable of breastfeeding. The belief that the tendency to have an insufficient milk supply is inherited from the mother has been noted previously [23]. It is possible that mothers who had not breastfed their daughters may find it particularly difficult to have their own daughters breastfeed successfully, as they believe this may reflect on their own abilities to nurture their infants.

Family support is an important factor in establishing successful breastfeeding and lack of family support may discourage it [1,21]. Identifying these issues in families may help the clinician address them during antenatal care and support the mother by reassuring female relatives that the capacity to breastfeed depends on the support and advice one receives and not on heredity.

Perceived characteristics of breast milk are another important determinant of breastfeeding duration [3]. The belief that a mother can harm her infant through her breast milk can be an important source of distress for the mother. Many women may abandon breastfeeding because they are afraid of hurting their baby. "Bad" milk, abdominal cramps, medications, and the maternal diet are all viewed as potential sources of harm for the infant.

The belief that abdominal cramps can be transferred from mother to infant through breast milk was surprising to the authors as they had not heard of it before the study and there is no mention of such a belief in the literature. This belief reflects that breastfeeding is not only about transmission of nutrition from mother to child, but also about transmission of physical pain. Given that abdominal cramping postpartum is essentially universal, due to uterine involution, this belief can be a very important barrier to breastfeeding. Understanding that this is a commonly held belief and addressing it before discharge from the hospital may be an important step towards improving a woman's chance of breastfeeding successfully.

Colostrum is seen as harmful in many cultures and discarding it is a common practice [5,11,16]. However, we are not aware of such a belief in Lebanese culture and did not see this concept emerge through the telephone calls to the hotline. Other causes of "bad milk" cited in the literature such as sexual intercourse, pregnancy, and working in the sun were not mentioned by our callers [13,15]. This may have been because the hotline was only available for four months postpartum. Had it been available for longer, these kinds of questions may have emerged.

The concept of the "kabseh" is an example in which breastfeeding relates to other people in society, not only the mother and infant. The belief that the evil eye could harm a mother's milk has been described in the literature [11]. In Egypt, the belief that the entrance of a menstruating woman into the room can harm a mother or baby is referred to as "mushahra" [11]. In Anatolia, not allowing another lactating woman to enter the house is believed to protect the mother and baby from evil forces [15]. The perception of the evil eye presents a barrier to women breastfeeding, because a mother might deny her child the benefits of her breast milk if she fears she has been subjected to the evil eye.

Our findings revealed that women might hold a number of beliefs that discourage them from breastfeeding successfully. Clinicians should attempt to elicit such beliefs and address them during the clinical encounter as a means to reassure mothers and encourage them to continue breastfeeding. Local and international organizations working to encourage breastfeeding, such as La Leche League, should also consider these beliefs when planning their programs.

In this study, the researchers did not specifically set out to investigate beliefs and traditions associated with breastfeeding. Women were not interviewed regarding their beliefs. Concerns that were expressed spontaneously in calls to the hotline were recorded. Therefore, common practices and beliefs would not have been identified if the callers did not specifically ask about them. There may be beliefs that are very common and so widely accepted that women do not question them or ask about them. Further studies that address this issue by asking women about their beliefs and practices could provide valuable information. Randomized trials could test the effectiveness of interventions to reduce maternal anxiety about breast milk quantity and quality.

## Conclusion

Several studies have shown that health care providers play an important role in a woman's decision to breastfeed [1,9,10,16]. Understanding local beliefs and customs that may influence breastfeeding can help direct clinicians to provide more culturally appropriate counselling about breastfeeding. Although the researchers live and provide healthcare for patients in Lebanon, they were not aware of many of the beliefs and practices that were discovered through this study. Studies that investigate beliefs and practices within other communities around the world could assist midwives, physicians and lactation specialists in providing more culturally sensitive care to their patients. This information could help clinicians encourage women to initiate and maintain breastfeeding.

## Competing interests

The authors declare that they have no competing interests.

## Authors' contributions

HO participated in the original study design, the data analysis, and drafting of the manuscript. LEZ conducted the data analysis and contributed to the writing of the manuscript. LW participated in interpretation of the data and revision of the manuscript. All authors read and approved the final manuscript.
